# Monocyte‐Derived Dendritic Cells: An Updated View on an Old Concept

**DOI:** 10.1111/imr.70075

**Published:** 2025-11-16

**Authors:** Elodie Segura

**Affiliations:** ^1^ Institut Necker Enfants Malades—INEM, CNRS, INSERM Université Paris Cité Paris France

**Keywords:** dendritic cells, human, monocyte, mouse

## Abstract

Dendritic cells comprise several populations with distinct ontogeny that share core features including a typical dendritic morphology and the ability to present antigens and stimulate T cells. Dendritic cells originating from monocytes have been reported in steady‐state and in different inflammatory contexts, in mouse models and in human clinical samples. However, because of their phenotypical and transcriptional proximity with other dendritic cell subsets and with monocyte‐derived macrophages, whether monocyte‐derived dendritic cells (mo‐DCs) represent a distinct population has been controversial. Here, we summarize the evidence supporting the existence of mo‐DCs in vivo and we review work addressing the molecular regulation of mo‐DCs differentiation and their role in immune responses. We also discuss the potential for harnessing mo‐DCs differentiation for therapy.

## 1 Introduction

Dendritic cells (DCs) are characterized by their ability to present antigens to T cells in lymphoid organs [1]. Other defining features include a typical morphology with dendrite‐like extensions and the capacity to migrate from peripheral tissues to draining lymph nodes [1]. Numerous studies in mouse and human have revealed DCs heterogeneity, and DCs subsets have been classified based on their ontogeny into classical DCs type 1 (cDC1), classical DCs type 2 (cDC2s), plasmacytoid DCs, transitional DCs and DC3s. Monocyte‐derived DCs (mo‐DCs) have also been described in mouse and human.

Monocytes circulate in the blood and can enter mucosal tissues in steady‐state and in virtually all tissues upon inflammation. Once in tissues, monocytes generally differentiate into macrophages, but in some cases were shown to generate DC‐like cells [2]. The ability of purified human monocytes to differentiate into antigen‐presenting cells in vitro in GM‐CSF culture has long been appreciated [3]. However, the existence of such mo‐DCs in vivo in tissues has remained controversial. Initial descriptions of mo‐DCs in vivo were mostly based on their mixed DC‐monocyte phenotype and assessed using adoptive transfer of monocytes [4]. In recent years, the advent of single‐cell omics and the development of novel fate‐mapping tools have challenged this concept on one hand, and provided novel insight into their ontogeny and function on the other hand. The discovery of DC3s [5, 6], displaying an intermediate cDC2/monocyte phenotype has further complicated the matter. However, accumulating evidence indicates that DC3s derive from a dedicated bone marrow progenitor, distinct from monocytes, and therefore do not represent mo‐DCs [7, 8, 9]. Based on recent studies from our laboratory and others, we propose a model whereby monocyte differentiation is dictated by microenvironmental signals and mo‐DCs participate in the division of labor between DCs subsets by presenting antigens in tissues rather than in lymphoid organs.

## 2 What Is the Evidence for the Existence of Mo‐DCs In Vivo?

Mo‐DCs were first described in mice during inflammation, and identified based on their expression of typical DCs markers (MHC class II and CD11c) together with “monocyte‐related” molecules including Ly6C, CD64, CCR2 or CD115 (complete list of markers reviewed in [2, 4]). Mo‐DCs were also described in steady‐state mucosal tissues, including skin [10], Peyer's patches [11], and peritoneal cavity [12]. In particular, morphological analyses revealed typical dendrites on their surface, similar to those of canonical DC [10, 12].

The discovery that cDC2s can upregulate some of these phenotypic markers upon exposure to inflammatory cues has cast some doubt on the identity of such cells in inflammatory contexts [13]. However, studies demonstrating the differentiation into antigen‐presenting cells of adoptively transferred monocytes have provided solid evidence for the existence of bona fide mo‐DCs (reviewed in [4]). Computational analyses have also been used to analyze DCs heterogeneity and infer their origin; however the absence of a monocyte signature can be misleading as transcriptional programs are impacted by inflammation. This is illustrated by a recent study using a radiotherapy model in which tumor‐infiltrating mo‐DCs and cDC2s were found to transcriptionally converge after treatment, making it impossible to distinguish them based on transcriptional signatures [14]. Fate‐mapping with a fluorescent reporter mouse demonstrated that most myeloid DCs were actually monocyte‐derived after treatment [14].

In humans, we and others have reported mo‐DCs in clinical samples both in steady‐state and inflammation. Human mo‐DCs are generally identified based on phenotype (expressing HLA‐DR, CD11c, CD1c, CD14, CD1a, CD88 and negative for CD163) and a monocyte‐like transcriptional signature [2]. In addition, morphological analyses have also shown the characteristic dendrites [15, 16, 17, 18, 19, 20, 21]. Mo‐DCs were identified in broncho‐alveolar lavage of healthy volunteers [16], non‐diseased vaginal mucosa [18, 19], peritoneal dialysis lavage [21], non‐inflamed peritoneal lavage [22], non‐diseased intestine [23, 24, 25], Peyer's patches [26] and non‐diseased anogenital mucosa [15]. In disease contexts, human mo‐DCs were also identified in inflamed lamina propria of Crohn's disease patients [25], skin from atopic dermatitis and psoriasis patients [27], pleural effusions from tuberculosis patients [28], synovial fluid from rheumatoid arthritis patients [29], peritoneal tumor ascites [20, 29], breast tumors [17] and lung tumors [30, 31].

This body of work provides solid evidence that monocytes can differentiate in vivo into cells with typical features of DCs. Whether referred to as monocyte‐derived antigen‐presenting cells, DC‐like cells, antigen‐presenting phagocytes, or mo‐DCs, these cells exist in a variety of contexts in both mouse and human.

## 3 Are all Monocytes Able to Differentiate Into DCs?

Recent studies using scRNA‐seq and fate‐mapping fluorescent reporter mouse strains have shown that classical monocytes (Ly6C^high^ CD43^low^) are generated through two distinct and parallel pathways: a majority from Granulocyte‐Monocyte Progenitors (GMPs) and a lower proportion (< 20%) from Monocyte‐Dendritic cell Progenitors (MDPs) [32, 33]. GMP‐derived monocytes are characterized by the expression of neutrophil‐related molecules, including CD177, elastase (encoded by *Elane*) and chitinase‐like protein 3 (encoded by *Chil3*), and can be traced based on their history of expression of *Ms4a3* [32, 34]. MDP‐derived monocytes express markers such as CD209a, CD319 (encoded by *Slamf7*), CD301b (encoded by *Mgl2*) and high levels of MHC class II molecules [32, 33].

Single‐cell high‐throughput analyses of human classical monocytes (CD14^high^ CD16^low^) have also revealed heterogeneity at the phenotypic and transcriptional levels [5, 6]. The neutrophil marker CXCR1 was proposed to characterize the human equivalents of GMP‐derived monocytes [35]. Of note, in human blood the CXCR1^+^ monocytes represented a minority of classical monocytes (< 30%) [35]. These observations suggest that the two distinct pathways of monocyte ontogeny are conserved between mouse and human but their respective contributions to the peripheral monocyte pool may differ between species, which could be due to different environmental signals.

Whether monocytes from both ontogenies differentiate into mo‐DC may depend on the context. Using the Ms4a3‐tdTomato reporter mouse, it was initially claimed that monocytes did not give rise to DCs in vivo, based on the lack of Tomato^+^ DCs in the steady‐state tissues analyzed [34]. However, in these mice, MDP‐derived monocytes and their progeny are not labeled, and inflammatory contexts were not analyzed. When placed in culture with GM‐CSF ex vivo, only MDP‐derived monocytes were found to differentiate into DC‐like cells [33, 36]. By contrast, in a model of cancer radiotherapy, GMP‐derived monocytes (traced using the Ms4a3‐tdTomato reporter strain) were shown to be the main source of mo‐DCs after treatment, which correlated with the levels of GM‐CSF [14]. However, GM‐CSF is not the only growth factor supporting mo‐DCs, as shown by the unaltered presence of mo‐DCs during inflammation in mice deficient for the GM‐CSF receptor [37, 38]. Further work is therefore needed to better understand in which contexts MDP‐derived and GMP‐derived monocytes can or cannot differentiate into DCs in vivo. In particular, GMP‐derived monocytes were found to be enriched upon systemic exposure to LPS [32, 33], but whether they can differentiate into mo‐DCs in such an inflammatory context was not investigated. Concerning human monocytes, this question remains unexplored.

## 4 What Factors Drive the Differentiation of Monocytes Into DCs?

Studies employing different model systems have shown that monocyte fate is not predetermined, but instead is modulated by external cues. In a classical model of human monocytes cultured with GM‐CSF, IL‐4 signaling was shown to trigger the DCs differentiation program [39, 40] via the action of the transcription factor NCOR2 [40]. In this model, mTORC1 inhibition was also shown to promote mo‐DCs differentiation [41]. To address the molecular regulation of monocyte differentiation, we established a refined in vitro differentiation system, in which human monocytes are cultured with M‐CSF, TNF and IL‐4 and give rise to both DCs and macrophages in the same culture wells [12]. Using this approach, we demonstrated that monocyte fate can be oriented by a variety of microenvironmental signals that remodel transcriptional networks. We evidenced an essential role for the ligand‐activated transcription factor aryl hydrocarbon receptor (AhR), showing that AhR activation promoted mo‐DCs differentiation by increasing expression of IRF4 and BLIMP‐1, while AhR inhibition promoted macrophage development driven by *MAFB* [12]. We also found that AhR activity controlled IRF4 expression by modulating the phosphorylation of STAT6 [42], which is activated downstream of IL4‐receptor, confirming the potency of IL‐4 signaling for inducing mo‐DCs differentiation. Using this differentiation model, we also uncovered a role for the transcription factors *MAFF* and *ZNF366* in promoting mo‐DCs differentiation [43]. In another study, we examined the impact of pathogen‐derived products. We found that mycobacteria and agonists of NOD receptors favored mo‐DCs differentiation at the expense of macrophages, via the action of TNF autocrine secretion [44]. TNF induced in monocytes the expression of miR‐155, which targets MAFB for degradation [44]. By contrast, exposure to viruses and agonists of TLR2, TLR4 or TLR7/8 promoted macrophage development over mo‐DCs, via mTORC1 activation and increased MAFB expression [44]. Finally, we showed that the transcriptional repressors ETV3 and ETV6 were required for mo‐DCs differentiation by repressing the expression of MAFB [45].

These findings were confirmed in mouse models. The central role of IRF4 in inducing a DC program, including antigen presentation, was also evidenced in culture models with GM‐CSF and IL‐4 [46, 47], as well as in vivo in IRF4‐deficient mice that lack serous cavities mo‐DCS in steady state [48]. We also showed using AhR‐deficient mice that AhR was required for steady‐state differentiation of mo‐DCs in skin and peritoneum [12]. Interestingly, steady‐state mo‐DCs were unperturbed in mice deficient for ETV6 in monocytes, but mo‐DCs differentiation was severely impaired during inflammation [45]. A similar observation was made in mice deficient for Nr4a3, a transcription factor proposed to act downstream of IRF4 [49]. This suggests that the in vivo requirements for mo‐DCs differentiation could be different according to the context, in particular in homeostasis versus inflammation.

Consistent with the idea that monocyte fate is modulated by external signals, we found that a diet supplemented with AhR agonists promoted mo‐DCs differentiation in steady‐state skin and peritoneum [12]. In addition, intra‐dermal injection of a TLR2 agonist promoted monocyte differentiation into macrophages, while injection of TNF specifically increased mo‐DCs development in the skin [44]. In the context of tumors, retinoic acid produced by tumor cells was proposed to favor monocyte differentiation towards macrophages by repressing IRF4 expression, which could be manipulated to increase mo‐DCs differentiation in tumors [50]. Finally, in a different pre‐clinical model, mo‐DCs were found to be largely absent from tumors but massively increased after radiotherapy [14].

**FIGURE 1:**
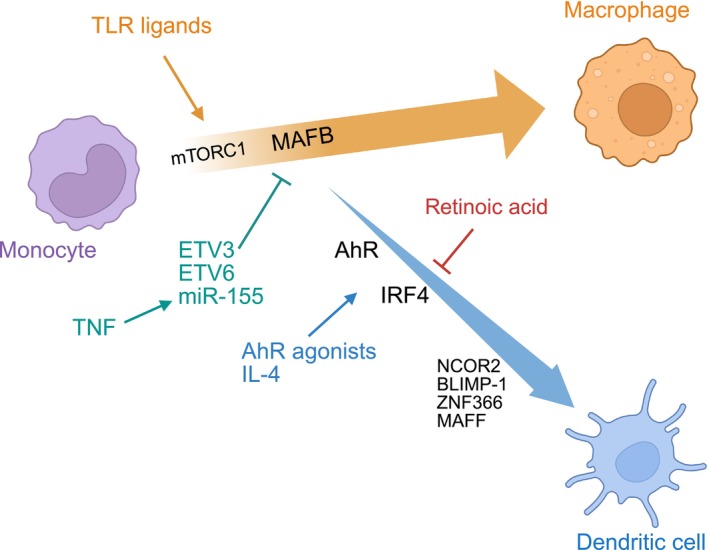
Proposed model for monocyte differentiation into macrophages versus dendritic cells. Monocytes differentiate into macrophages by a default pathway promoted by the activation of mTORC1 and MAFB expression. Microbe sensing via TLRs activates mTORC1 and favors macrophage differentiation. Upon exposure to TNF, monocytes upregulate miR‐155, ETV3 and ETV6 which can actively repress MAFB expression thereby licensing monocytes for dendritic cell differentiation. The dendritic cell differentiation program is induced by the activation of AhR and IL‐4 signaling, resulting in the expression of IRF4. Retinoic acid can inhibit IRF4 expression in tumors and favor macrophage differentiation. Other transcription factors involved in dendritic cell differentiation include NCOR2, BLIMP‐1, ZNF366 and MAFF.

Based on these findings, we propose that monocytes differentiate into macrophages via a default program that needs to be actively repressed at the transcriptional level in order to allow for the induction of the mo‐DCs fate commitment (Figure 1). Work from our laboratory and others indicates that this mo‐DCs pathway can be induced by a variety of external cues, enabling monocytes to rapidly adapt to signals from their micro‐environment.

## 5 What Is the Function of Mo‐DCs?

The ability to migrate from peripheral tissues to lymph nodes is considered a key feature of DCs [1]. However, in vivo mouse studies suggest that mo‐DCs have a low migratory capacity. In several studies, migratory cells with a phenotype consistent with mo‐DCs could not be detected in lymph nodes [51, 52, 53, 54]. Poor migratory capacity was also evidenced after monocyte adoptive transfer during acute lung inflammation [55]. In a lung infection model, only inflammatory cDC2 were found to migrate to draining lymph nodes, but not mo‐DCs [13]. Consistent with this, mo‐DCs were shown to present antigens to T cells directly in tissues rather than in lymphoid organs. In a seminal study based on experimental virus reactivation using HSV1, virus‐specific CD4^+^ and CD8^+^ T cells were found to proliferate locally without recirculating, which was abolished upon monocyte depletion using clodronate liposomes [56]. This study was the first to suggest that mo‐DCs could stimulate locally tissue‐resident memory T cells. In a model where skin was first inflamed using a chemical agent and subsequently infected with HSV1, mo‐DCs were shown to present a viral antigen to activated virus‐specific CD4^+^ T cells directly in the skin [57]. This phenomenon was impaired in CCR2‐deficient mice, in which monocytes cannot egress from the bone marrow, confirming that the antigen‐presenting cells were of monocyte origin. During influenza infection in reporter mouse models in which monocytes and monocyte‐derived cells are fluorescent, imaging showed that mo‐DCs interacted with antigen‐specific CD8^+^ T cells directly in the lung [58] and in the trachea [54]. The use of CCR2‐deficient mice also confirmed the monocyte origin of these DCs.

Mo‐DCs were also identified in lymph nodes during experimental autoimmune encephalomyelitis (EAE) [38, 45, 59], which is induced by the injection of a myelin antigen together with mycobacteria‐derived products. By deleting MHC class II molecules specifically in monocyte‐derived cells, mo‐DCs were shown to be the main antigen‐presenting cells in the draining lymph nodes [59]. We also found that mo‐DCs were decreased in mice deficient for ETV6 in monocytes, which correlated with reduced induction of antigen‐specific CD4^+^ T cells in the draining lymph nodes [45]. In this model, lymph nodes mo‐DCs could derive from monocytes directly recruited to the lymph nodes from the blood.

The functional properties of human mo‐DCs have been analyzed ex vivo. Mo‐DCs isolated from clinical samples were found to efficiently stimulate naive CD4^+^ T cells in allogeneic assays, polarizing them towards Th17 cells via the secretion of IL‐23 for mo‐DCs from pleural effusions from tuberculosis patients [28] and from peritoneal tumor ascites [29], or Th1 cells for intestinal mo‐DCs [23]. We and others also showed that dermal mo‐DCs isolated from non‐diseased skin induced Tfh polarization [60, 61]. Using mo‐DCs isolated from peritoneal tumor ascites, we also demonstrated that mo‐DCs had the ability to cross‐present antigens via a non‐canonical lysosomal pathway, to secrete IL‐12p70 and to induce the differentiation of effector cytotoxic CD8^+^ T cells [20]. Whether antigen presentation takes place directly in tissues remains challenging to address in a human context.

Collectively, these studies support the concept that mo‐DCs would play a role complementary to that of cDCs, by acting as peripheral antigen‐presenting cells. This would allow the restimulation of effector T cells that have been primed by cDCs in lymph nodes or the rapid activation of tissue‐resident memory T cells directly in situ.

## 6 Should Mo‐DCs be Targeted In Vivo for Therapy?

Because of their central role in numerous inflammatory pathologies and in cancer, monocytes have been the target of a variety of therapeutic strategies aiming at blocking their recruitment or their differentiation into macrophages. The specific targeting of mo‐DCs or the modulation of monocyte differentiation into macrophages versus DCs remains in its infancy.

In inflammatory disorders, human mo‐DCs were proposed to fuel the inflammation by stimulating pathogenic T cells and secreting deleterious inflammatory mediators, in particular in rheumatoid arthritis and Crohn's disease [29, 62, 63]. This notion was directly demonstrated in the EAE mouse model, where mo‐DCs played a central role in inducing myelin‐specific CD4^+^ T cells [45, 59] and in the secretion of IL1‐β in the inflamed central nervous system [38]. Consistent with this, when mo‐DCs differentiation was blocked by the deletion of ETV6 in monocytes, mice were protected from disease development [45]. Blocking mo‐DCs differentiation while preserving that of monocyte‐derived macrophages may be preferable to shutting down monocyte recruitment to the inflamed tissue. Indeed, monocyte‐derived macrophages are involved in tissue repair in lesions, as evidenced in particular during the resolution phase of EAE [64, 65, 66].

In the context of cancer therapy, the role of mo‐DCs remains unclear and could depend on the cancer type and treatment. In a pioneer study in mice treated with chemotherapy, mo‐DCs were shown to present antigens directly in the tumor, which was proposed to be the main presentation source as surgical removal of draining lymph nodes did not decrease CD8^+^ T cell activation [67]. Another study showed that inhibition of retinoic acid signaling in monocytes promoted the differentiation of mo‐DCs in the tumor and correlated with improved responses to anti‐checkpoint therapy [50]. These findings suggest that skewing monocyte differentiation towards mo‐DCs development could promote treatment efficacy. By contrast, mo‐DCs that developed following tumor radiotherapy were shown to mediate treatment resistance by inducing T regulatory cells [14]. More work will be needed to understand the context‐dependent role of mo‐DCs in anti‐tumoral responses, and to design adequate therapeutic strategies.

## 7 Conclusion and Perspectives

The development of single‐cell technologies, high‐throughput transcriptomics and fate‐mapping genetic mice models has considerably advanced our understanding of the differentiation and functional properties of monocyte‐derived cells in tissues. Accumulating evidence indicates that monocyte differentiation is influenced by a wide range of external cues, including cytokines, danger signals and metabolites such as retinoic acid and AhR‐activating indole derivatives. This may underlie important differences between mouse and human tissues, due to varying exposure to environmental factors.

A number of questions remain to be addressed before we can harness monocyte differentiation and mo‐DCs properties for therapy. In particular, the role of mo‐DCs in cancer remains to be fully elucidated and might be highly context‐dependent. A better understanding of the molecular regulators of monocyte fate commitment and transcriptional reprogramming will be essential for identifying novel targets for manipulating monocyte differentiation. The impact on mo‐DCs differentiation of trained immunity, which can be induced in humans in particular through vaccination, will also deserve further attention. Blocking mo‐DCs while preserving macrophages may be beneficial in T cell‐driven inflammatory pathologies and some types of anti‐cancer treatments. Finally, the role of mo‐DCs remains underexplored in some pathological contexts where this knowledge could provide novel therapeutic opportunities, such as allograft rejection and granulomatous diseases.

## Author Contributions

E.S. wrote the manuscript.

## Conflicts of Interest

The author declares no conflicts of interest.

## Data Availability

The author have nothing to report.
